# Identification of novel KIF11 mutations in patients with familial exudative vitreoretinopathy and a phenotypic analysis

**DOI:** 10.1038/srep26564

**Published:** 2016-05-23

**Authors:** Jia-Kai Li, Ping Fei, Yian Li, Qiu-Jing Huang, Qi Zhang, Xiang Zhang, Yu-Qing Rao, Jing Li, Peiquan Zhao

**Affiliations:** 1 Department of Ophthalmology, Xin Hua Hospital Affiliated to Shanghai Jiao Tong University School of Medicine, 1665 Kong Jiang Road, Shanghai, 200092, China

## Abstract

*KIF11* gene mutations cause a rare autosomal dominant inheritable disease called microcephaly with or without chorioretinopathy, lymphedema, or mental retardation (MCLMR). Recently, such mutations were also found to be associated with familial exudative vitreoretinopathy (FEVR). Here, we report 7 novel *KIF11* mutations identified by targeted gene capture in a cohort of 142 probands with FEVR who were diagnosed in our clinic between March 2015 and November 2015. These mutations were: p.L171V, c.790-2A>C, p.Q525*, p.Q842*, p.S936*, p.L983fs and p.R1025G. Phenotypic analysis revealed that all of the affected probands had advanced FEVR (stage 4 or above). Three had microcephaly, and one had chorioretinopathy, which indicated a phenotypic overlap with MCLMR. Two mutations were also found in the families of the affected probands. One parent with a p.R1025G mutation had an avascular peripheral retina and abnormal looping vessels. However, one parent with p.L983fs had normal retina, which indicated incomplete penetration of the genotype. Our results further confirmed that *KIF11* is causative of FEVR in an autosomal dominant manner. We also suggest the examination of MCLMR-like features, such as microcephaly, chorioretinopathy, for patients with FEVR and wide-field fundus photography for patients with MCLMR in future practice.

*KIF11* (NM_004523, gene id: 3832) encodes a mitotic kinesin also known as Eg5. Eg5 has long been recognized as an important member of the kinesin-like protein family and is involved in the development of malignant cancer and angiogenesis^1^. Consequently, Eg5 is regarded as one of the most promising new targets for antimitotic drugs^2^. This protein has three distinctive functional domains: a microtubule-binding motor region, a stalk region, and a tail region. In 2012, mutations in *KIF11* were found to be associated with the development of a rare autosomal dominant inheritable disease called microcephaly with or without chorioretinopathy, lymphedema, or mental retardation (MCLMR) (OMIM 152950). The disease was first described by Jarmas *et al.* in 1981^3^ and subsequently by Crowe and Dickerman in 1986^4^. As the name indicates, patients with this disease often display a variable spectrum of central nervous system and ocular developmental anomalies. Thus far, 45 pathological mutations of *KIF11* have been associated with MCLMR^5^,^6^,^7^,^8^,^9^,^10^.

Familial exudative vitreoretinopathy (FEVR) is an inheritable disorder of retinal blood vessel development that leads to the incomplete vascularization of the retina and poor vascular differentiation^11^. This condition was first described by Criswick and Schepens in 1969^12^. The clinical manifestations of this disease are complicated and variable. Mild forms of the disease can be asymptomatic and only exhibit peripheral retinal vascular abnormalities, such as a peripheral avascular zone, venous telangiectasias and altered arterial tortuosity. Severe forms of FEVR are associated with retinal neovascularization, subretinal and intraretinal hemorrhages, exudates, retinal folds and tractional retinal detachment^13^. Thus far, approximately 50% of the clinically identified patients with FEVR have been found to be associated with the following four genes in the Wnt signaling pathway: *NDP*, *FZD4*, *LRP5,* and *TSPAN12*^14^,^15^. The critical roles of the Wnt pathway in ocular development and retinal vascular development have also been demonstrated in various tissue-specific Wnt pathway gene knockout mice^16^. Additionally, mutations in *ZNF408* have also been found in FEVR patients^17^.

The association between *KIF11* mutations and FEVR was first reported in November 2014^18^. This study identified 5 *KIF11* mutations in 72 screened FEVR probands and concluded that the mutations were inherited in an autosomal dominant manner. This study was followed by another recently published study that identified 4 novel mutations in a cohort of 48 FEVR patients^19^. These results indicate that *KIF11* may be another important gene that is involved in the development of FEVR.

In this study, we report 7 novel *KIF11* mutations in FEVR patients which were identified through targeted gene capture, and we analyze the clinical phenotypes associated with these mutations.

## Results

### Cohort description and mutation detection rate

Between March 2015 and November 2015 we identified 142 FEVR probands based on clinical presentation from among the patients who came to our clinic. There were 87 males and 55 females. The medium age was 34 months, and the range was 1.5 months to 53 years old. None of the patients had a history of premature birth.

Targeted gene capture followed by next-generation sequencing (NGS) was performed on 142 probands. Initial sequencing was performed using a custom retinal disease capture panel that included the following FEVR-related genes: *NDP*, *FZD4*, *LRP5*, *TSPAN12*, *ZNF408,* and *KIF11*. Pathological mutations, as judged by various analytical approaches described in the Methods, were further validated by Sanger sequencing in each proband and his/her parents and siblings (if any). We identified 52 probands with pathogenic mutations in the *NDP*, *FZD4*, *LRP5*, *TSPAN12* and *ZNF408* genes (36.62%) and 7 probands with novel mutations in *KIF11* (4.93%). For the detection of the *KIF11* mutations, the average sequencing depth was 389.94. The average coverage of the target region was 99.47%. Moreover, the average coverage of the targeted exons for >10X reads was 96.14% and that for >20X reads was 91.72%.

### New *KIF11* mutations and the associated clinical presentations

Seven novel *KIF* mutations were detected in 7 patients. The nature of the mutations, the predicted pathogenicities and the clinical presentations of the probands are listed in Table 1. With the exception of one patient (Patient No. 3), all were diagnosed with FEVR for the first time in our clinic.

We identified the following five *de novo* mutations: c.511C>G (p.L171V), c.790-2A>C, c.1573C>T (p.Q525^*^), c.2524C>T (p.Q842*), and c.2807C>G (p.S936*). All of these mutations were heterozygous for the respective sites. The affected probands all exhibited clinically advanced FEVR.

**Figure 1:**
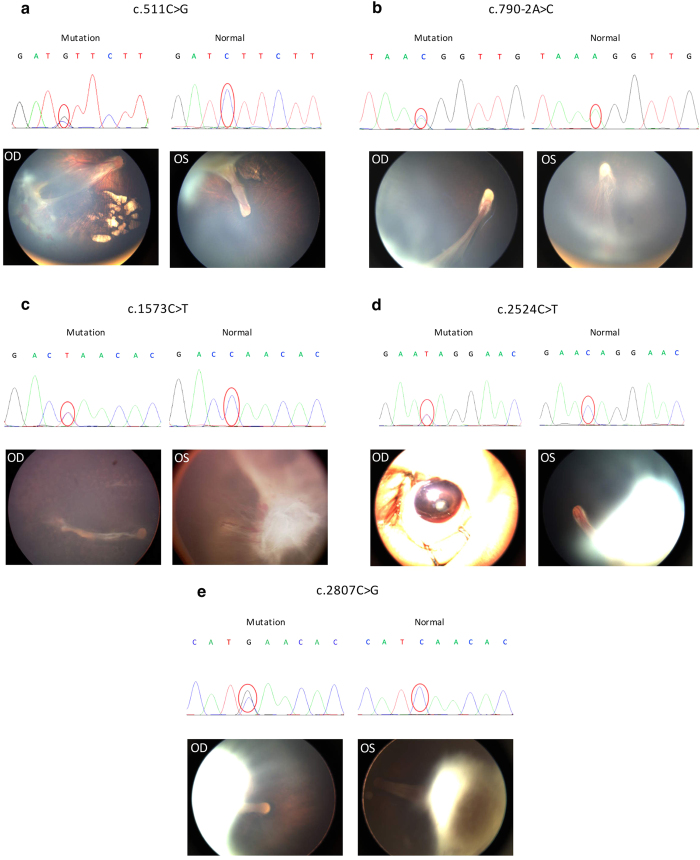
Chromatograms of five *de novo* mutations and the corresponding fundus pictures of the affected probands. The sequence variations are marked with red circles. OD and OS represent right and left eyes, respectively. (**a**) chromatograms and fundus pictures of a 4-month-old male with the c.511C>G (p.L171V) mutation. (**b**) chromatograms and fundus pictures of a 1-year-old female with the splicing mutation c.790-2A>C. (**c**) chromatograms and fundus pictures of a 7-year-old female with c.1573C>T (p.Q525*) mutation. (**d**) chromatograms and fundus pictures of a 10-month-old female with a c.2524C>T (p.Q842*) mutation. (**e**) chromatograms and fundus pictures of a 1-year-old male with a c.2807C>G (p.S936*) mutation. Notice the existence of chorioretinopathy in panel a (both eyes); the falciform retinal folds in panels a (OS), b (OD), c (OD), d (OS) and e (both eyes); the retrolenticular fibrotic masses in panels c (OS), d (OS) and e (both eyes); cataracts and microphthalmia in panel d (OD); dragged disc and macular in panels a (OD) and b (OS).

The carrier of the c.511C>G (p.L171V) mutation was a 4-month-old male. He was referred to our clinic for the inability to follow moving objects. A fundus examination revealed stage 4 FEVR with chorioretinopathy in both eyes. Inferotemporal dragging of the optic disc and macula by the fibrovascular mass was observed in the right eye. A retinal detachment involving the macula was observed in the left eye (Fig. 1a). At the age of 1 year, his head circumference was 39 cm, which was 6 standard deviations (SDs) below the mean.

The splicing mutation c.790-2A>C affected the splicing of exons 7–8. The carrier was a 1-year-old female who exhibited a typical falciform retinal fold with a retrolenticular fibrotic mass in the right eye and an inferotemporal fibrotic proliferation with a dragged optic disc in the left eye (Fig. 1b). At the age of 2 years, her head circumference was 44 cm, which was 2 SDs below the mean.

The carrier of the c.1573C>T (p.Q525^*^) mutation was a 7-year-old girl. She had undergone panretinal photocoagulation therapy in her right eye prior to attending our clinic and presented with a retinal fold. The left eye exhibited total retinal detachment and microphthalmia (Fig. 1c). Her head circumference was normal.

The carrier of the c.2524C>T (p.Q842^*^) mutation was a 10-month-old female. She presented with total retinal detachment, cataracts and a shallow anterior chamber in the right eye and advanced FEVR features in the left eye that included retinal folds and a retrolenticular fibrotic mass (Fig. 1d). Her head circumference was 43 cm when she was 18 months old, which was 2 SDs below the mean. Additionally, she was diagnosed with mild mental retardation.

The carrier of the c.2807C>G (p.S936^*^) was a 1-year-old male. He exhibited bilateral falciform retinal detachment with a retrolenticular fibrotic mass (Fig. 1e). His head circumference was normal.

**Figure 2:**
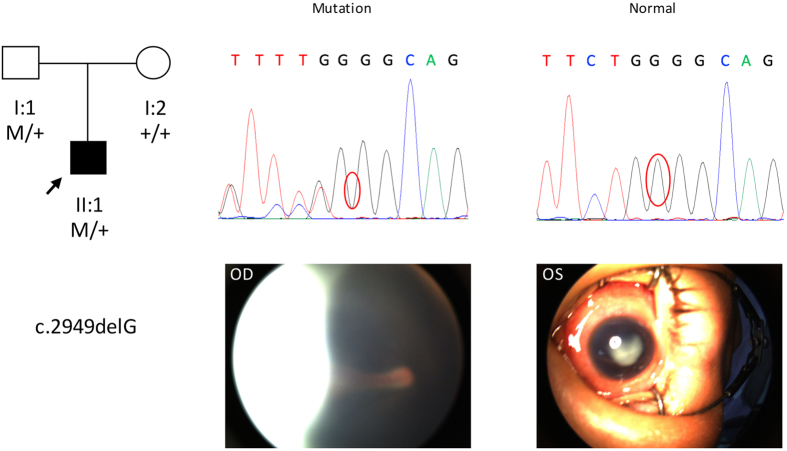
Pedigree, chromatograms and fundus pictures of the frameshift mutation c.2949delG (p.L983fs). In the pedigree, M sign represents a variant; +, a normal allele. In the chromatograms, the variation is marked with red cycle. OD and OS represent right and left eyes, respectively. This mutation was detected in a 2-year-old boy and it was inherited from his father. Fundus examination showed bilateral FEVR with retinal folds. The microphthalmic left eye had cataracts and fibrotic mass which obstructed retinal imaging.

The frameshift mutation c.2949delG (p.L983fs) was detected in a 2-year-old boy who was heterozygous for the mutation and inherited it from his father. The boy exhibited bilateral FEVR with retinal folds and a fibrotic mass that obstructed the retinal imaging of the left eye (Fig. 2). However, we did not observe any retinal abnormality via fluorescein angiography in the father who also carried the mutation. The head circumference of this boy was normal.

**Figure 3:**
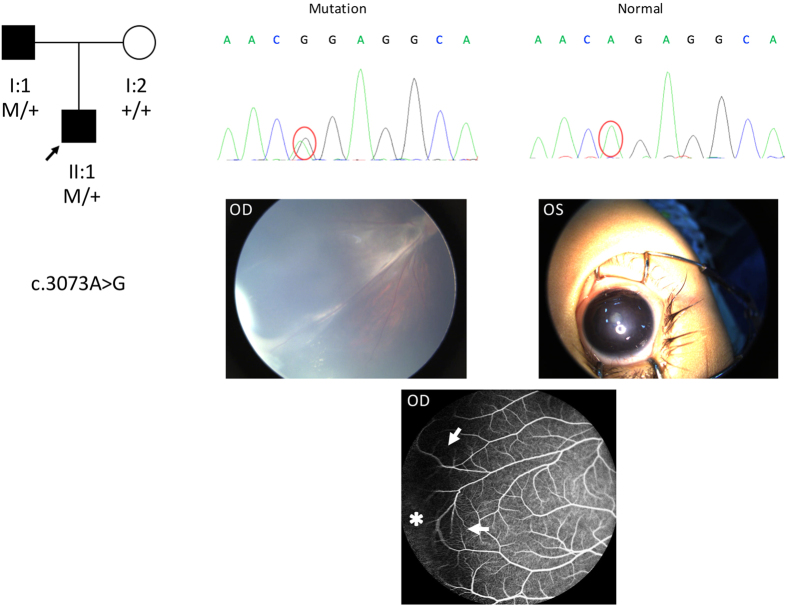
Pedigree, chromatograms and fundus pictures of the missense mutation c.3073A>G (p.R1025G). In the pedigree, M sign represents a variant; +, a normal allele. In the chromatograms, the variation is marked with red cycle. OD and OS represent right and left eyes, respectively. This mutation was detected in a 1-year-old boy and it was inherited from his father. Fundus examination showed a peripheral fibrovascular proliferation with exudates in the right eye and microphthalmia with cataracts in the left eye. Fundus fluorescein angiography of his father (bottom) revealed an avascular peripheral retina (asterisk) and vascular looping in the right eye (arrows).

Another missense mutation, i.e., c.3073A>G (p.R1025G), was detected in a 1-year-old boy. This mutation was also detected in his father. The boy exhibited peripheral fibrovascular proliferation with exudates in the right eye and microphthalmia with cataracts in the left eye. His head circumference was normal. Fundus fluorescein angiography of his father revealed an avascular peripheral retina and vascular looping in the right eye (Fig. 3).

## Discussion

**Figure 4:**
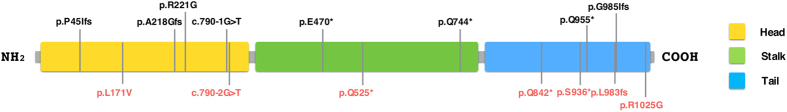
Location of the reported and new *KIF11* mutations associated with FEVR. Mutations found in this study are labeled in red at the bottom. Mutations reported earlier are labeled in black on the top^17^,^18^. The three functional domains are depicted in different colors as labeled.

Despite the extensive research on the protein product of *KIF11,* mutations of this gene and their associations with hereditary diseases were only recently discovered. A literature search identified 6 studies that reported approximately 45 mutations involved in MCLMR^5^,^6^,^7^,^8^,^9^,^10^ and 2 studies that reported 9 *KIF11* mutations associated with FEVR^18^,^19^. One mutation was found in both MCLMR and FEVR patients. These two studies on FEVR patients reported the *KIF11* mutation detection rates of 6.94% (5 out of 72 screened) and 8.33% (4 out of 48 screened), respectively^18^,^19^. Here, we reported the identification of 7 additional novel heterozygous mutations in *KIF11* in 142 screened FEVR probands, which represents a mutation detection rate of 4.93% among all of the FEVR patients. The present study is by far the largest cohort study that has screened for *KIF11* mutations in FEVR patients. The distribution of *KIF11* mutations found thus far in the FEVR patients is presented in Fig. 4. A similar summary of the mutations found in MCLMR patients is also available^10^. In both diseases, approximately 40% of the mutations are located in the motor region, 20% in the stalk region and 40% in the tail region.

Unlike in MCLMR, the hallmarks of which are both central nervous system and ocular developmental defects, the primary defect of FEVR is limited to abnormal retinal vasculature. The shared clinical features of advanced FEVR and MCLMR include retinal folds, retinal detachment, cataracts and microphthalmia. Chorioretinopathy and optic nerve atrophy are not typical presentations of FEVR^20^. In the seven FEVR probands who carried *KIF11* mutations, we observed a phenotypic overlap between these two diseases. We identified 3 probands with microcephaly, 1 with chorioretinopathy, 1 with mild mental retardation, and 3 with microphthalmia (Table 1). A similar phenotypic overlap has also been reported previously by two groups^18^,^19^. At the protein level, these findings are consistent with the function of the KIF11 protein. *In vivo* studies in zebra fish and chicken embryos have revealed that the KIF11 protein is predominantly expressed in lymphoblasts and endothelial cells. Defective KIF11 protein causes developmental and vascular defects^21^. An interesting question to pursue is why some *KIF11* mutations cause broader defects that manifest as MCLMR while others cause relatively focused diseases such as FEVR.

Incomplete penetrance and variable expressivity of the *KIF11* mutations were also observed in the affected FEVR patients. Among the two inherited heterozygous mutations (p.L983fs and p.R1025G) found in this study, one parent (with a p.R1025G mutation) exhibited a mild clinical phenotype, whereas the other parent (with a p.L983fs mutation) appeared to be normal. Variable expressivity of the *KIF11* mutations was demonstrated by c.790-2A>C. A similar mutation (c.790-1G>T), which also affects the splicing of exons 7–8, has been previously reported by two separate groups. This mutation caused FEVR in one patient and MCLMR in the other patient^8^,^18^. Both mutations were *de novo*. All three of the probands had microcephaly. However, the ocular abnormalities in the MCLMR patient were different than those in the two FEVR patients. Between the two FEVR patients, it appeared that the patient in our study had a milder presentation than the one reported by Robitaille *et al.*^18^. However, because our patient was younger and it was her first diagnosis of the disease, it is possible that the condition may progress to greater severity.

Overall, we noticed that the 7 probands carrying the *KIF11* mutations identified in this study all had advanced FEVR; three had stage 4, and four had stage 5 FEVR. This association between severe FEVR phenotypes and *KIF11* mutations has also previously been observed by another group^18^. This association also seems to differ from the clinical presentations associated with *NDP*, *FZD4*, *LRP5* and *TSPAN12* gene mutations, which display broader phenotypic spectra and primarily vary from stage 2 to stage 5. However, additional studies are needed to conclude whether mutations of *KIF11* have more profound pathogenic effects in FEVR.

In conclusion, we identified 7 novel *KIF11* mutations during the screening of 142 FEVR patients. FEVR due to *KIF11* mutations represents a mechanism that leads to incomplete development of the retinal vasculature that differs from the well-known mechanisms related to the Wnt signaling pathway. Combined with the findings from other groups, the existing evidence suggests that *KIF11* mutations in FEVR patients are often associated with severe clinical manifestations, although incomplete phenotype penetration and variation exist. We suggest that FEVR patients in the clinic undergo examinations for typical MCLMR features, such as microcephaly, chorioretinopathy, lymphedema, and mental retardation and that MCLMR patients undergo wide-field fundus photography. We also suggest the inclusion of the *KIF11* gene in future genetic screens for FEVR.

## Methods

### Study subjects and clinical data collection

This study was approved by the Institutional Review Board of Xin Hua Hospital affiliated to the Shanghai Jiao Tong University School of Medicine. All work was performed in accordance with the approved study protocol. Informed written consent was obtained from the parents or guardians of each participant because they were all under-aged children.

The FEVR probands were identified from among the patients who attended our clinic between March 2015 and November 2015 based on the presence of at least one of the following retinal vascular developmental anomalies as previously described^22^: a lack of peripheral retinal vascular development with or without variable degrees of nonperfusion, vitreoretinal traction, subretinal exudation, retinal neovascularization occurring at any age or total retinal detachment with a fibrotic mass behind the lens. All participants were born full-term. The disease severities of all probands were further classified according to the staging system described by Ranchod *et al.*^20^. Each proband was subjected to the following ocular examinations: wide-field fundus photography using either a RetCam (Clarity Medical Systems, Pleasanton, CA, USA) or an Optos 200Tx (Optos, Inc., Marlborough, MA, USA), and indirect ophthalmoscopy with a 28D lens and scleral depression when needed. Additionally, their direct family members, primarily the parents, were subjected to fundus fluorescein angiography using Spectralis HRA2 (Heidelberg Engineering GmbH, Germany).

### Targeted gene capture and next-generation sequencing (NGS)

Targeted gene capture and sequencing were performed by MyGenostics (MyGenostics, MD, USA). Briefly, peripheral blood was drawn from each proband and their direct family members, and the genomic DNA was extracted and fragmented. Illumina adapters were added to the fragments, and the samples were size-selected for the 350–400 bp products. This pool of DNA fragments was amplified using PCR and allowed to hybridize with DNA capture probes that were specifically designed for the targeted genes. The captured DNA fragments were eluted, amplified again and subjected to NGS using an Illumina HiSeq 2000 (Illumina, Inc., San Diego, CA, USA). A custom Genetic Pediatric Retinal Diseases Panel based on targeted exome capture technology was used and covered the following twenty-one genes: *ABCB6, GDF6, LRP5, RS1, SOX2, TENM3, VSX2, FZD4, IKBKG, NDP, SALL2, STRA6, TSPAN12, YAP1, GDF3, KIF11, PAX6, SHH, TBX1, TUBA8,* and *ZNF408*.

### Data analysis

The sequenced reads were mapped to the UCSC hg19 (http://genome.ucsc.edu) human reference genome using the Burrows Wheeler Aligner (BWA) (http://bio-bwa.sourceforge.net/bwa.shtml). Variants were detected with GATK and further annotated using the 1000 Genomes database, ESP6500, dbSNP and the company’s own in-house database of 800 samples. The pathogenicity of each variant was assessed with the following databases: PolyPhen-2 (http://genetics.bwh.harvard.edu/pph2/), Sorting Intolerant From Tolerant (http://sift.jcvi.org/www/SIFT_enst_submit.html), Mutation Taster (http://www.mutationtaster.org/), and GERP++ (http://mendel.stanford.edu/SidowLab/downloads/gerp/index.html). For the autosomal dominant mutations, a reported minor allele frequency of more than 0.01 according to the Exome Aggregation Consortium (http://exac.broadinstitute.org/gene/ENSG00000138160) or benign variants predicted by both PolyPhen-2 and SIFT were excluded. The remaining variants were considered as pathogenic candidates and were further searched in the HGMD database to determine whether they had been reported previously as pathogenic.

### PCR and Sanger sequencing validation

Primer3 was used to design all of the PCR primers for the Sanger sequencing that was conducted to validate the potential pathogenic variants. The average amplicon size was 400 bp. The DNA was sequenced on the ABI 3130XL platform and subsequently analyzed using Mutation Surveyor.

## Additional Information

**How to cite this article**: Li, J.-K. *et al.* Identification of novel KIF11 mutations in patients with familial exudative vitreoretinopathy and a phenotypic analysis. *Sci. Rep.* **6**, 26564; doi: 10.1038/srep26564 (2016).

## Figures and Tables

**Table 1:** Novel *KIF11* mutations identified in FEVR patients.

Patient No.	Sex	Age at diagnosis	cDNA change#	Amino acid change	Location	MAF	*In silico* analysis score				FEVR stage	Other clinical findings
							SIFT	Poly-Phen2	Mutation-Taster	GERP++		
1	M	4 months	c.511C>G	p.L171V	exon 5	NA	D	PD	Dc	C	4	Chorioretinopathy, microcephaly (-6 SDs)
2	F	12 months	c.790-2A>C	Splicing	exon 8	NA	NA	NA	Dc	C	4	Microcephaly (-2 SDs)
3	F	7 years	c.1573C>T	p.Q525*	exon 13	NA	T	NA	Dc	NC	5	Microphthalmia in the left eye
4	F	10 months	c.2524C>T	p.Q842*	exon 18	NA	T	NA	Dc	C	5	Microcephaly (-2 SDs), mild mental retardation, microphthalmia in the right eye
5	M	12 months	c.2807C>G	p.S936*	exon 20	NA	T	NA	Dc	C	5	Microphthalmia in the left eye
6	M	2 years	c.2949delG	p.L983fs	exon 21	NA	NA	NA	NA	NA	5	None
7	M	12 months	c.3073A>G	p.R1025G	exon 22	0.0002&	D	B	P	NC	5	None

^#^Coding sequence change was based on DNA reference sequence NM_004523.

^&^This frequency was reported by 1000 genomes database Aug 2015 version. MAF: minor allele frequency, checked against 1000 genomes database, ESP6500 and in-house database of 800 entries. NA: not available. Pathogenic results were shown as “D (damaging)” for SIFT, “PD (probably damaging)” for Polyphen2, “Dc (disease-causing)” for Mutation Taster, and “C (conserved)” for GERP++. Non-pathogenic results were shown as “T (tolerated)” for SIFT, and “B (benign)” for Polyphen2, “P(Polymorphism)” for Mutation Taster, and “NC (Nonconserved)” for GERP++.
